# IL-13 and skin fibrosis in inflammatory skin diseases

**DOI:** 10.3389/fimmu.2026.1974049

**Published:** 2026-09-24

**Authors:** Noriko Ikegawa, Tan Manh Nguyen, Natsuko Saito-Sasaki, Yu Sawada

**Affiliations:** 1 Department of Dermatology, University of Occupational and Environmental Health, Kitakyushu, Japan; 2 Department of Dermatology, Hanoi Medical University, Hanoi, Vietnam; 3 Department of Dermatology, National Hospital of Dermatology and Venereology, Hanoi, Vietnam

**Keywords:** fibrosis, IL-13, mechanism, skin, skin diseases

## Abstract

Skin fibrosis is increasingly recognized as an important component of chronic inflammatory skin diseases, contributing to dermal thickening, tissue stiffness, and persistent functional impairment. Although transforming growth factor-β (TGF-β) has long been considered the central mediator of fibrosis, accumulating evidence indicates that interleukin-13 (IL-13), a key cytokine of type 2 immunity, plays a pivotal role in linking chronic inflammation to fibrotic remodeling. Unlike classical scar formation, IL-13-associated fibrosis is characterized by sustained immune activation, continuous extracellular matrix remodeling, and residual tissue plasticity, suggesting that it represents a dynamic and potentially reversible process. In this review, we summarize the current understanding of the mechanisms by which IL-13 promotes skin fibrosis. We discuss direct activation of fibroblasts through the IL-13Rα1/STAT6 pathway, dysregulation of extracellular matrix synthesis and degradation, neuroimmune interactions involving chronic itch and mechanotransduction, macrophage–fibroblast crosstalk, and emerging evidence for fibroblast memory mediated by epigenetic remodeling. Furthermore, we compare the pathological features of IL-13-associated fibrosis across representative skin diseases, including atopic dermatitis, prurigo nodularis, systemic sclerosis, and keloids. This conceptual framework provides a unified perspective on the diverse roles of IL-13 in cutaneous fibrosis and highlights the importance of integrating immunological, mechanical, and stromal mechanisms.

## 1 Introduction

Fibrotic remodeling of the skin is increasingly recognized as a clinically relevant component of chronic inflammatory dermatoses (1). Beyond visible skin thickening and stiffness, fibrosis compromises cutaneous elasticity, leading to persistent cutaneous functional impairment (2, 3). Importantly, once established, fibrotic changes are often poorly responsive to conventional anti-inflammatory therapies, highlighting the need to better understand the mechanisms that link chronic inflammation to structural remodeling of the skin (4, 5).

Skin fibrosis has traditionally been discussed primarily in the context of transforming growth factor-β (TGF-β)–driven wound healing and scarring (6). However, accumulating evidence suggests that fibrotic remodeling in inflammatory skin diseases cannot be fully explained by canonical profibrotic pathways alone (7). In particular, type 2 immune responses, which dominate conditions such as atopic dermatitis (AD) and prurigo, are increasingly implicated in the development and persistence of fibrotic skin changes (8).

Interleukin-13 (IL-13) is a central effector cytokine of type 2 immunity and is abundantly expressed in lesional skin of type 2–dominant inflammatory dermatoses. While IL-13 is well established as a driver of inflammation, its contribution to fibrotic remodeling of the skin has only recently gained attention (9). Notably, IL-13 exerts sustained effects within the cutaneous microenvironment, influencing fibroblast activation, extracellular matrix dynamics, and epidermal–dermal interactions.

From an evolutionary perspective, IL-13–mediated type 2 immune responses can be viewed as a conserved tissue-protective strategy (10). By promoting tissue remodeling and reinforcement, IL-13-driven pathways likely conferred a survival advantage by limiting damage and invasion under hostile environmental conditions (8). However, persistent activation of this reparative response may contribute to chronic fibrotic remodeling in inflammatory skin diseases.

In this review, we focus on the mechanisms by which IL-13 links type 2 inflammation to fibrotic remodeling in the skin, discuss its role across inflammatory dermatoses, and consider the therapeutic implications of targeting IL-13–driven fibrosis.

Importantly, the relative contribution of IL-13 to fibrosis differs among inflammatory skin diseases. In some settings, IL-13 may directly promote fibroblast activation and extracellular matrix production, whereas in others, particularly systemic sclerosis and keloids, it may function predominantly as an amplifier within broader profibrotic networks in which TGF-β and other pathways remain major drivers.

## 2 Fibrosis as an adaptive tissue-protective response

Although fibrosis is generally regarded as a pathological consequence of chronic inflammation, transient fibrotic remodeling may represent an evolutionarily conserved tissue-protective response that reinforces tissue integrity following injury or infection (7, 11). Type 2 immunity, with IL-13 as a central effector cytokine, contributes to this reparative program by coordinating immune activation with fibroblast-mediated extracellular matrix remodeling and tissue repair (12–15).

However, persistent activation of this normally protective response can promote pathological fibrosis. In atopic dermatitis and prurigo nodularis, sustained type 2 inflammation may maintain IL-13 signaling and extracellular matrix remodeling beyond the period required for tissue repair (14–17). Thus, IL-13-associated fibrosis can be viewed, at least in part, as persistence of a physiological repair program beyond its normal resolution.

## 3 The source of IL-13 in the skin and its mechanisms of action

In the skin, IL-13 is mainly produced by immune cells, particularly group 2 innate lymphoid cells (ILC2s) and Th2 cells (18, 19). In addition to ILC2s, other innate immune cells, including mast cells, eosinophils, and basophils, can contribute to the type 2 cytokine milieu and may serve as additional sources of IL-13 under inflammatory conditions (19). These cells may participate in fibrotic remodeling not only through IL-13 production but also through the release of other inflammatory and profibrotic mediators that influence fibroblast activation and extracellular matrix remodeling. Thus, IL-13-associated skin fibrosis likely reflects coordinated interactions among adaptive and innate immune cells rather than the activity of a single cellular source.

Secreted IL-13 binds to two receptors, IL-13 receptor alpha 1 (IL-13Rα1) and IL-13 receptor alpha 2 (IL-13Rα2, 20). IL-13Rα1 forms a functional receptor complex through association with IL-4Rα, leading to intracellular signal transduction via the JAK–STAT6 pathway and the induction of downstream inflammatory responses in the skin (21). In contrast, IL-13Rα2 lacks a substantial intracellular signaling domain and has therefore been regarded as a so-called decoy receptor (22, 23). In the skin, the precise functional role of IL-13Rα2 remains unclear; however, its expression is upregulated under inflammatory conditions, suggesting a potential role in scavenging excess IL-13 and contributing to the resolution of acute inflammation. Supporting this notion, studies using cell lines expressing IL-13Rα2 alone have shown that IL-13 can be internalized in a receptor-dependent manner and subsequently degraded in lysosomes, indicating that IL-13Rα2 may function as a regulatory sink for IL-13 in cutaneous inflammation (22).

Although IL-13 is the primary focus of this review, its signaling should be considered in the context of IL-4, another major type 2 cytokine. IL-4 and IL-13 share the type II receptor complex composed of IL-4Rα and IL-13Rα1 and consequently activate overlapping JAK–STAT6 signaling pathways. Thus, some profibrotic responses attributed to IL-13, including fibroblast activation and alternatively activated macrophage polarization, may also be induced or reinforced by IL-4. Nevertheless, IL-13 has distinct receptor biology, particularly through its interaction with IL-13Rα2, which does not bind IL-4 and can regulate IL-13 availability and signaling. Moreover, accumulating evidence suggests a prominent role for IL-13 in tissue remodeling and fibrosis. This distinction is therapeutically relevant because selective IL-13 inhibition and dual IL-4/IL-13 pathway blockade may differentially modulate inflammatory and fibrotic components of type 2–mediated skin disease.

## 4 Mechanistic basis of IL-13–induced skin fibrosis

IL-13–associated skin fibrosis represents a slowly evolving remodeling process that develops under chronic type 2 inflammation, rather than as an abrupt, end-stage scarring event. In contrast to TGF-β–dominant fixed fibrosis, IL-13–driven fibrosis remains closely linked to ongoing immune activity, extracellular matrix turnover, and tissue mechanics, accounting for its dynamic and potentially reversible nature (**Figure 1**).

**Figure 1:**
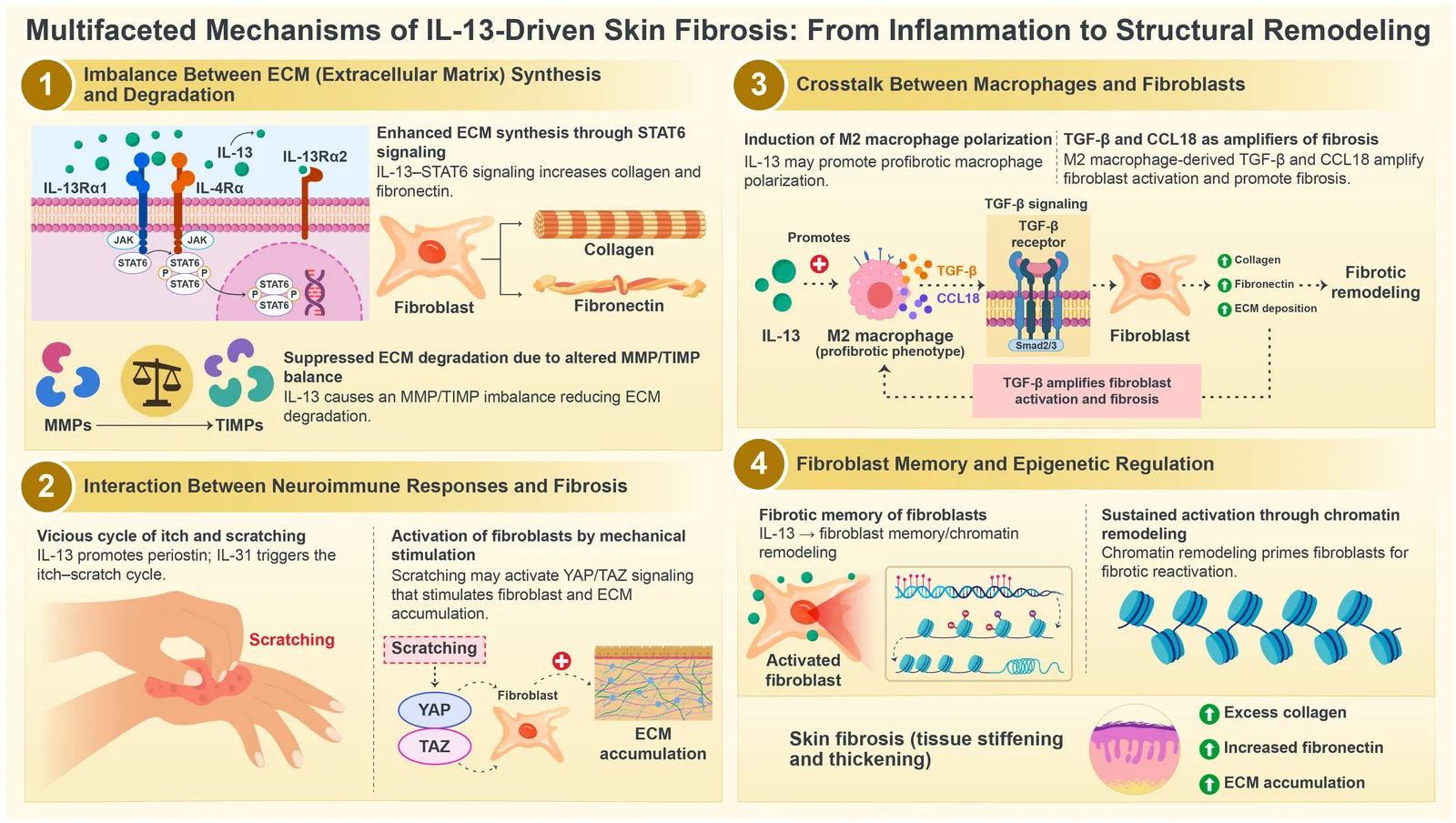
Proposed mechanisms of IL-13-associated skin fibrosis in inflammatory skin diseases. IL-13 promotes cutaneous fibrotic remodeling through multiple interconnected mechanisms. IL-13 directly activates dermal fibroblasts via the IL-13Rα1/IL-4Rα–STAT6 pathway, increasing collagen and fibronectin synthesis while reducing extracellular matrix turnover through an imbalance between matrix metalloproteinases (MMPs) and tissue inhibitors of metalloproteinases (TIMPs). IL-13 also contributes to chronic itch and mechanical stress associated with type 2 inflammation, thereby promoting repetitive scratching and mechanotransduction that further enhance fibroblast activation. In addition, IL-13 induces alternatively activated macrophages, which produce profibrotic mediators such as TGF-β and CCL18, thereby reinforcing macrophage–fibroblast crosstalk. Finally, prolonged IL-13-driven inflammation may establish fibroblast memory through epigenetic remodeling, facilitating recurrent profibrotic gene expression. Collectively, these interconnected pathways promote extracellular matrix accumulation, dermal thickening, and progressive fibrotic remodeling in chronic inflammatory skin diseases. Solid lines indicate relatively well-established mechanisms, whereas dashed lines indicate emerging or proposed mechanisms for which direct evidence in cutaneous fibrosis remains limited.

### 4.1 Extracellular matrix synthesis/degradation imbalance driven by IL-13

In IL-13-associated fibrosis, extracellular matrix accumulation reflects not only enhanced matrix synthesis but also impaired matrix turnover. IL-13 signals through the IL-13Rα1/IL-4Rα receptor complex to activate STAT6 in fibroblasts, inducing a profibrotic transcriptional program characterized by increased expression of collagen and fibronectin (24). In parallel, IL-13 shifts the balance toward reduced matrix degradation by altering the expression of matrix metalloproteinases and their endogenous inhibitors, thereby promoting extracellular matrix accumulation (15).

Beyond its direct effects on matrix production and degradation, IL-13 also modifies fibroblast responsiveness to profibrotic cues. In IL-13-rich inflammatory environments, particularly in type 2–dominant diseases such as AD, IL-13 may contribute to profibrotic remodeling in part through interactions with TGF-β signaling, thereby reinforcing extracellular matrix accumulation and tissue remodeling (15). In this context, IL-13 may function as an amplifier of profibrotic signaling rather than as an independent dominant driver. Collectively, these effects favor extracellular matrix accumulation and progressive stabilization within the dermis, leading to gradual tissue thickening and loss of elasticity.

### 4.2 Neuroimmune–fibrotic interactions in IL-13–associated skin fibrosis

In addition to its direct effects on fibroblasts, IL-13 may promote skin fibrosis through neuroimmune mechanisms that connect chronic itch, scratching, and mechanical tissue injury. This pathway is particularly relevant in prurigo nodularis and lichenified atopic dermatitis, in which persistent pruritus and repeated scratching are closely associated with dermal thickening and fibrotic remodeling.

IL-13 contributes to chronic itch by acting on both immune and non-immune components of the skin (25, 26). Type 2 inflammation increases the production of pruritogenic mediators, including IL-31 and periostin, and enhances the sensitivity of cutaneous sensory nerves. Periostin, which is induced under IL-13–rich conditions, can stimulate sensory neurons and amplify itch signaling, whereas IL-31 directly activates pruriceptive pathways (27, 28). Neurogenic mediators released from activated sensory nerves, such as substance P and calcitonin gene-related peptide (CGRP), may further reinforce local inflammation and vascular activation, thereby sustaining a tissue environment permissive to fibrosis (29).

Importantly, itch itself may function as a profibrotic stimulus. Repeated scratching produces chronic mechanical injury, activates keratinocytes, disrupts the epidermal barrier, and induces the release of epithelial cytokines and profibrotic mediators (25). These signals can repeatedly re-engage IL-13–primed fibroblasts and promote extracellular matrix deposition. Mechanical stress generated by scratching may also activate mechanotransduction pathways, including integrin-dependent signaling and YAP/TAZ activation, thereby linking external mechanical trauma to persistent fibroblast activation (30). Although mechanotransduction through integrin and YAP/TAZ signaling is well established as a mechanism of fibroblast activation in fibrotic tissues, direct evidence linking this pathway to IL-13-associated cutaneous fibrosis remains limited. Thus, its contribution in inflammatory skin diseases should currently be regarded as an emerging mechanism requiring further validation in skin.

Suppression of itch may provide benefits beyond symptomatic relief by reducing repetitive scratching and chronic skin injury. Whether such effects translate into attenuation of dermal fibrotic remodeling remains to be determined.

### 4.3 Macrophage–fibroblast crosstalk in IL-13–associated skin fibrosis

Although IL-13 directly activates dermal fibroblasts through STAT6 signaling, accumulating evidence indicates that macrophages serve as critical intermediaries that amplify and sustain fibrotic remodeling. Rather than acting independently, macrophages and fibroblasts establish a reciprocal cellular network in which immune-derived signals are translated into persistent extracellular matrix remodeling.

IL-13 promotes the differentiation and activation of alternatively activated macrophages, which acquire a profibrotic phenotype characterized by the production of TGF-β and CCL18 (14, 31–33). These mediators stimulate fibroblast proliferation, enhance myofibroblast differentiation, and promote extracellular matrix deposition. Thus, macrophages function as signal amplifiers that convert transient type 2 immune activation into sustained stromal remodeling.

Emerging evidence from fibrotic tissues suggests reciprocal interactions between macrophages and fibroblasts, in which macrophage-derived profibrotic mediators activate fibroblasts, while activated fibroblasts remodel the extracellular matrix and alter the local microenvironment, thereby facilitating persistent profibrotic responses (34, 35). Whether an analogous macrophage–fibroblast circuit operates in IL-13-associated inflammatory skin diseases remains to be determined. Importantly, much of the evidence supporting this macrophage–fibroblast feed-forward circuit has been derived from fibrotic organs other than the skin, and direct evidence for an equivalent IL-13–TGF-β circuit in cutaneous fibrosis remains limited.

Recent single-cell transcriptomic studies have highlighted the remarkable heterogeneity of macrophages in fibrotic tissues (36). Rather than representing a uniform M2 population, profibrotic macrophages comprise multiple transcriptionally distinct subsets, including osteopontin (SPP1)-expressing macrophages, which interact closely with activated fibroblasts in several fibrotic organs (37). Although the precise identity of these macrophage subsets in inflammatory skin diseases remains to be fully defined, similar macrophage–fibroblast communication networks may contribute to IL-13-associated skin fibrosis; however, this possibility requires direct validation in cutaneous tissues.

Collectively, these findings suggest that IL-13–associated fibrosis should be viewed as the consequence of coordinated multicellular interactions rather than direct fibroblast activation alone. Targeting macrophage–fibroblast communication, in combination with IL-13 blockade, may therefore represent a promising therapeutic strategy for interrupting chronic fibrotic remodeling while preserving physiological tissue repair.

### 4.4 Fibroblast memory and epigenetic remodeling in skin fibrosis

Persistent inflammatory signals can induce long-lasting functional changes in fibroblasts, termed fibroblast memory (38). Chronic IL-13 exposure may contribute to this process through epigenetic mechanisms, including histone modifications, DNA methylation, and microRNA regulation such as miR-135b (12, 38, 39).

Importantly, fibroblast memory is unlikely to depend solely on IL-13–STAT6 signaling. Mechanical signals generated by extracellular matrix stiffness can sustain fibroblast activation through integrin/FAK and YAP/TAZ pathways, while epigenetic mechanisms stabilize persistent transcriptional states (30, 38). Thus, fibroblast memory may integrate inflammatory, epigenetic, and mechanical signals, potentially contributing to the chronicity and recurrence of fibrotic remodeling.

## 5 IL-13 in type 2–mediated inflammatory skin diseases

On the basis of this mechanistic framework, IL-13–associated fibrosis manifests with distinct clinical phenotypes across type 2–mediated inflammatory skin diseases, reflecting differences in the degree of stromal stabilization and residual tissue plasticity (**Table 1**). Although IL-13 contributes to fibrotic remodeling across these conditions, its relative pathogenic importance and fibrotic consequences are not uniform. In diseases such as atopic dermatitis and prurigo, IL-13 is persistently enriched within lesional skin, creating a localized cytokine environment that sustains fibroblast activation and matrix retention without progressing to fixed, scar-like fibrosis.

**Table 1:** IL-13-associated fibrotic remodeling across representative skin diseases.

Disease	Principal IL-13 source(s)	Degree/nature of fibrosis	Role of IL-13	Relevant therapeutic evidence
Atopic dermatitis (9, 45, 46)	Th2 cells, ILC2s	Mild-to-moderate, inflammation-associated dermal remodeling; relatively dynamic and potentially reversible	Direct contributor to fibroblast activation and ECM remodeling in an IL-13-rich environment	Selective IL-13 inhibition improves clinical disease activity; direct reversal of established fibrosis has not been demonstrated
Prurigo nodularis (47–54).	Th2 cells, ILC2s and other type 2 immune cells	Moderate-to-marked dermal remodeling with fibroblast activation and ECM accumulation; strongly influenced by chronic scratching	Amplifies fibrosis through type 2 inflammation, fibroblast activation, and the itch–scratch cycle	IL-4Rα/IL-13 pathway inhibition improves disease; improvement in remodeling may occur indirectly through suppression of itch and scratching
Systemic sclerosis (57, 59–65).	IL-13-producing CD8+ T cells, particularly during the early inflammatory phase	Severe, progressive, relatively fixed fibrosis with reduced tissue plasticity	Primarily an amplifier within a TGF-β-dominant profibrotic network	Romilkimab targeting IL-4/IL-13 showed improvement in skin fibrosis in early diffuse cutaneous SSc; IL-13 blockade alone is unlikely to reverse established fibrosis
Keloid (69, 70).	Not clearly established; type 2 immune cells may contribute	Localized but highly persistent and mechanically stabilized scar fibrosis	Contributor/amplifier within a TGF-β- and mechanotransduction-dominant network; altered IL-13Rα2 may enhance responsiveness	Direct clinical evidence for IL-13-targeted therapy is currently limited

Importantly, this pattern is maintained by continuous local IL-13 availability rather than transient cytokine exposure, allowing fibrotic remodeling to persist independently of overt tissue injury. The resulting fibrotic features therefore remain dynamically coupled to inflammation and vary according to disease context. In the following sections, we discuss how IL-13–associated fibrosis is differentially expressed across representative type 2 inflammatory skin diseases, highlighting disease-specific manifestations of stromal stabilization, reversibility, and clinical remodeling.

## 6 AD

Although fibrosis is not a defining feature of AD, chronic lesions frequently exhibit lichenification, dermal thickening, and increased collagen deposition, reflecting substantial stromal remodeling associated with persistent type 2 inflammation (9, 40–43). IL-13 may contribute to this process by promoting fibroblast activation and altering extracellular matrix homeostasis.

Unlike systemic sclerosis or mature scars, dermal remodeling in AD appears to remain dynamically linked to inflammatory activity and may retain greater reversibility (44, 45). Clinical improvement with selective IL-13 inhibition supports the importance of ongoing type 2 inflammation, although such responses do not by themselves demonstrate direct reversal of established fibrosis (46).

## 7 Prurigo nodularis

Prurigo nodularis is a chronic pruritic disorder characterized by multiple hyperkeratotic nodules on the skin (47). The development of prurigo nodular lesions is closely linked to persistent itch, which promotes repeated scratching and creates a microenvironment favoring fibrotic remodeling driven by both external mechanical stress and internal pruritus-dominant conditions (48, 49).

Dermal fibrotic remodeling is a critical pathological feature of prurigo nodularis, with histological analyses demonstrating fibroblast activation and extracellular matrix accumulation within lesional skin (47, 50). Similar to AD, IL-13 is dominantly expressed in prurigo nodularis lesions (51). Clinical efficacy of IL-4Rα/IL-13 pathway-targeted therapy suggests that suppression of IL-13 signaling may facilitate improvement of fibrotic remodeling, likely through attenuation of itch-driven mechanical stress and fibroblast activation rather than direct antifibrotic effects (52, 53).

Recent single-cell RNA sequencing analyses have further highlighted the unique stromal microenvironment of prurigo nodularis (39, 51). Compared with AD, prurigo nodularis exhibits enhanced extracellular matrix organization, collagen biosynthesis, and expansion of profibrotic fibroblast subsets, indicating that stromal remodeling is a defining feature of the disease. These findings suggest that persistent type 2 inflammation in prurigo nodularis is accompanied by stable fibroblast activation and reciprocal interactions between immune and stromal cells, thereby reinforcing chronic tissue remodeling. Although IL-13 is unlikely to be the sole driver of fibrosis, it may function as an important amplifier linking chronic pruritus, neuroimmune activation, and dermal remodeling. Given the molecular and clinical heterogeneity of prurigo nodularis, reliable biomarkers that reflect the relative contributions of type 2 inflammation, neuroimmune dysregulation, and fibrotic remodeling are needed to better define disease endotypes and guide individualized therapeutic selection. In particular, biomarkers capable of predicting responses to therapies targeting IL-4/IL-13, IL-31, and other neuroimmune pathways may facilitate a more precise, mechanism-based approach to the management of prurigo nodularis (54).

## 8 Systemic sclerosis

SSc is characterized by progressive fibrosis, microvascular dysfunction, and immune activation (55–58). Although TGF-β remains a principal driver of fibrosis (59), IL-13 also contributes to disease pathogenesis (60, 61). IL-13-producing CD8+ T cells have been identified in SSc, particularly during the early inflammatory phase, providing a potential link between immune activation and dermal fibrosis (62). IL-13 can enhance collagen production and fibroblast responsiveness to TGF-β and promote profibrotic macrophage activation, thereby amplifying TGF-β-dominant fibrotic responses (61, 62).

Unlike the more inflammation-dependent remodeling observed in AD and prurigo nodularis, SSc fibrosis becomes stabilized through multiple profibrotic and mechanical pathways and progressively less dependent on type 2 inflammation (30, 63–67). Thus, IL-13 is better viewed as an amplifier rather than the dominant driver of established SSc fibrosis. Consistent with this concept, dual IL-4/IL-13 inhibition with romilkimab showed improvement in skin fibrosis in early diffuse cutaneous SSc, suggesting that targeting this pathway may be most relevant during inflammation-dominant stages (68).

## 9 Keloid

Keloids are characterized by persistent extracellular matrix deposition extending beyond the original wound and represent dysregulated wound healing in which tissue repair fails to terminate appropriately (69–71). Although TGF-β remains a principal profibrotic mediator, IL-13 also contributes to fibroblast activation and collagen production through STAT6 signaling (72). Notably, reduced IL-13Rα2 expression in keloid fibroblasts may enhance IL-13 responsiveness by removing a potential regulatory mechanism that limits IL-13 signaling (73).

IL-13 signaling may also cooperate with the abnormal biomechanical environment of keloids. Increased extracellular matrix stiffness and tension activate mechanotransduction pathways, including integrin/FAK and YAP/TAZ, potentially reinforcing fibroblast activation (30). Thus, IL-13 is likely to function as a contributor or amplifier within a broader network dominated by TGF-β, extracellular matrix stiffness, and persistent fibroblast activation rather than as an independent driver of keloid fibrosis.

Importantly, clinical improvement following IL-13- or IL-4Rα-targeted therapy should not necessarily be interpreted as evidence of direct reversal of established fibrosis. Improvements in skin thickening or fibrotic features may occur indirectly through suppression of inflammation and, particularly in prurigo nodularis, through interruption of the itch–scratch cycle and consequent reduction of repetitive mechanical injury. Therefore, direct antifibrotic effects of IL-13 pathway inhibition should be distinguished from secondary improvement in tissue remodeling resulting from control of the underlying inflammatory and mechanical stimuli.

## 10 Conclusions and future directions

Cutaneous fibrosis results from persistent interactions among immune, stromal, and mechanical pathways. Although IL-13 contributes to fibrotic remodeling across several inflammatory skin diseases, its relative importance differs by disease context, ranging from a direct contributor in type 2–dominant inflammatory dermatoses to an amplifier of broader profibrotic networks in systemic sclerosis and keloids.

Future studies should identify biomarkers that distinguish reversible from established fibrosis and clarify disease- and tissue-specific fibrotic mechanisms using approaches such as single-cell and spatial analyses. Therapeutically, targeting inflammation alone may be insufficient once persistent stromal activation is established. Antifibrotic agents such as nintedanib (74), epigenetic modulation (75), and JAK inhibition (76) represent potential complementary approaches for disrupting fibrosis-maintaining pathways.

Combining IL-13 pathway inhibition with strategies targeting persistent fibroblast activation is therefore an attractive concept, although such approaches remain largely hypothetical in cutaneous fibrosis and require further preclinical and clinical validation. Ultimately, effective therapies will need to suppress pathological remodeling while preserving physiological tissue repair.
